# Repressor and activator protein accelerates hepatic ischemia reperfusion injury by promoting neutrophil inflammatory response

**DOI:** 10.18632/oncotarget.8509

**Published:** 2016-03-30

**Authors:** Chang Xian Li, Chung Mau Lo, Qizhou Lian, Kevin Tak-Pan Ng, Xiao Bing Liu, Yuen Yuen Ma, Xiang Qi, Oscar Wai Ho Yeung, Vinay Tergaonkar, Xin Xiang Yang, Hui Liu, Jiang Liu, Yan Shao, Kwan Man

**Affiliations:** ^1^ Department of Surgery, The University of Hong Kong, Hong Kong, China; ^2^ Collaborative Innovation Center for Diagnosis and Treatment of Infectious Diseases, The University of Hong Kong, Hong Kong, China; ^3^ Shenzhen Institute of Research and Innovation, The University of Hong Kong, Hong Kong, China; ^4^ Department of Medicine, The University of Hong Kong, Hong Kong, China; ^5^ Institute of Molecular and Cellular Biology, Biopolis, Singapore

**Keywords:** liver transplantation, hepatic ischemia reperfusion injury, neutrophils migration, inflammatory response, nuclear factor-κB

## Abstract

Repressor and activator protein (Rap1) directly regulates nuclear factor-κB (NF-κB) dependent signaling, which contributes to hepatic IRI. We here intended to investigate the effect of Rap1 in hepatic ischemia reperfusion injury (IRI) and to explore the underlying mechanisms. The association of Rap1 expression with hepatic inflammatory response were investigated in both human and rat liver transplantation. The effect of Rap1 in hepatic IRI was studied in Rap1 knockout mice IRI model *in vivo* and primary cells *in vitro*. Our results showed that over expression of Rap1 was associated with severe liver graft inflammatory response, especially in living donor liver transplantation. The results were also validated in rat liver transplantation model. In mice hepatic IRI model, the knockout of Rap1 reduced hepatic damage and hepatic inflammatory response. In primary cells, the knockout of Rap1 suppressed neutrophils migration activity and adhesion in response to liver sinusoidal endothelial cells through down-regulating neutrophils F-Actin expression and CXCL2/CXCR2 pathway. In addition, the knockout of Rap1 also decreased production of pro-inflammatory cytokines/chemokines in primary neutrophils and neutrophils-induced hepatocyte damage. In conclusion, Rap1 may induce hepatic IRI through promoting neutrophils inflammatory response. Rap1 may be the potential therapeutic target of attenuating hepatic IRI.

## INTRODUCTION

Hepatic ischemia reperfusion injury (IRI) contributes to primary liver non-function and increases the incidence of acute and chronic rejection [1]. Furthermore, hepatic IRI also contributes to late phase tumor recurrence and metastasis after liver surgery for the patients with hepatocellular carcinoma [2–4]. Increasing evidence showed that hepatic IRI is a typical inflammatory response involving a complex web of interactions between various cellular and molecular signals [5, 6]. Toll-like receptor 4 (TLR4) and C-X-C motif chemokine 10 (CXCL10) activate the triggering mechanism of liver inflammation [7, 8]. Activated macrophage contributes to the generation of a host of proteins associated with inflammatory response and secretion of pro-inflammatory cytokines/chemokines [6, 9]. During hepatic IRI, neutrophils transmigrate into the liver parenchyma and generate lots of superoxide and hypochlorous acid, which can either directly diffuse into hepatocytes or induce intracellular oxidant stress [10, 11]. Furthermore, neutrophils-derived proteases are able to directly cause liver injury by aggravating inflammatory response [12]. The transcription factor NF-κB, a master regulator of inflammation and cell death, play central roles in the processes of inflammatory response during hepatic IRI [13]. Inhibition of neutrophils and NF-κB signaling effectively protected liver against IRI [9, 14].

The mammalian repressor and activator protein (Rap1, also known as telomeric repeat-binding factor 2-interacting protein 1), is a component of the shelterin complex at the telomere and dependent on TRF2 for stability and recruitment [15]. In contrast to other components of the shelterin complex, Rap1 is dispensable for telomere end capping and does not participate in non-homologous end joining mediated repair. Instead, Rap1 is essential to negatively regulate telomere recombination and is involved in regulation of homology directed repair [16]. Although shelterin components mainly function at telomeres, Rap1 also has non-telomeric functions. Mammalian Rap1 is recruited to non-telomeric (TTAGGG)_2_ DNA consensus motifs through interaction with the TRF2 or additional factors, and regulates gene expression [17–19]. Rap1 can protect from obesity through binding to PPARα and PGCα and modulates their transcription [20]. Recently, Teo H et al. demonstrated that Rap1 acts as a regulator of the NF-κB signaling through promoting IKK-mediated p65 phosphorylation, leading to the activation of NF-κB target genes expression [21]. Mice with knockout of Rap1 are resistant to endotoxic shock and decrease the expressions of inflammatory cytokines/chemokines in response to LPS. However, the effect of Rap1 in hepatic IRI is still unknown. Our hypothesis is that the inhibition or knockout of Rap1 may attenuate hepatic IRI by regulating liver inflammatory response.

In this project, we aim to study the effect of Rap1 in hepatic IRI and to explore the underlying mechanisms. The association of Rap1 expression with hepatic inflammatory response were investigated in both human and rat liver transplantation. The direct role of Rap1 in regulating hepatic IRI was explored in Rap1 knockout mice IRI model *in vivo* and primary cells *in vitro*.

## RESULTS

### Association study

#### Over expression of Rap1 was associated with severe hepatic inflammatory response after human liver transplantation

**Figure 1:**
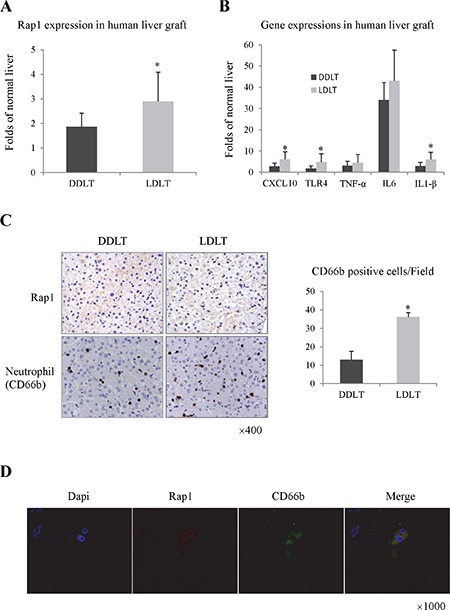
Over expression of Rap1 was associated with severe hepatic inflammatory response after human liver transplantation (**A**) The intragraft mRNA level of Rap1 at 2 hours after human transplantation. (**B**) The intragraft expressions of pro-inflammatory cytokines/chemokines at 2 hours after human transplantation. (**C**) The expressions of Rap1 and neutrophil (CD66b) in liver graft were detected by IHC staining. (**D**) The double staining of Rap1 and CD66b were detected by IF staining. The gene expression levels were calculated as folds of normal liver. (*P < 0.05).

In clinical samples, Rap1 was up-regulated in liver graft at 2 hours after portal vein reperfusion (Figure 1A and 1C). The expression of Rap1 in living donor liver transplantation (LDLT) was higher than that in diseased donor liver transplantation (DDLT) (Figure 1A and 1C). High intragraft expression of Rap1 was associated with more neutrophils infiltration, and higher expressions of pro-inflammatory cytokines/chemokines in liver graft (Figure 1B and 1C). Furthermore, over-expressed Rap1 was mainly found in neutrophils (Figure 1D).

#### Over expression of Rap1 was associated with severe hepatic inflammatory response after rat liver transplantation

Rap1 was up-regulated in liver graft at 4 hours after rat transplantation, especially in small-for-size (SFS) liver graft (Supplementary Figure 1A). Furthermore, over-expression of Rap1 was also associated with increased neutrophils infiltration and high expressions of inflammatory cytokines/chemokines after rat liver transplantation (Supplementary Figure 1B and 1C).

### *In vivo* functional study using Rap1 knockout mice

#### The knockout of Rap1 attenuated hepatic injury after major hepatectomy and partial hepatic IRI

**Figure 2:**
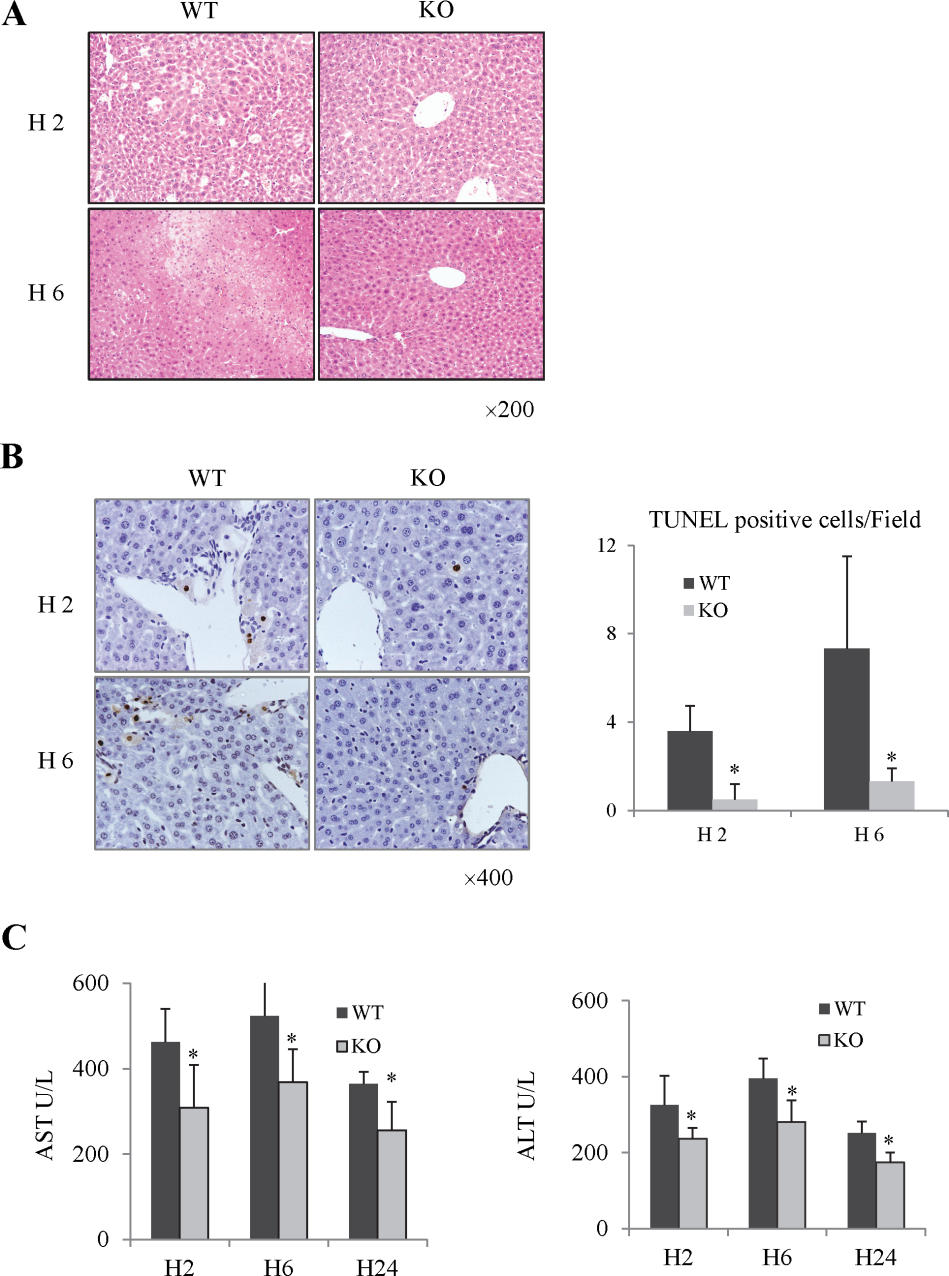
The knockout of Rap1 attenuated hepatic injury in mouse IRI model (**A**) Histological change was detected by H&E staining. (**B**) Cell apoptosis were detected by Tunnel staining. (**C**) Comparison of AST and ALT between Rap1 wild type and knockout group. (*Compared to wild type group *P < 0.05*; *N* = 5−6/group).

Rap1 was up-regulated in mouse liver after hepatic IRI (Supplementary Figure 2A and 2B). Compared to wild type group, hepatic lobular architecture and portal tracts were well preserved in Rap1 knockout group after IRI (Figure 2A). Furthermore, less apoptotic cells were found in Rap1 knockout group (Figure 2B). Serum levels of AST and ALT were also lower in Rap1 knockout group compared to wild type group (Figure 2C).

#### The knockout of Rap1 decreased hepatic inflammatory response after major hepatectomy and partial hepatic IRI

**Figure 3:**
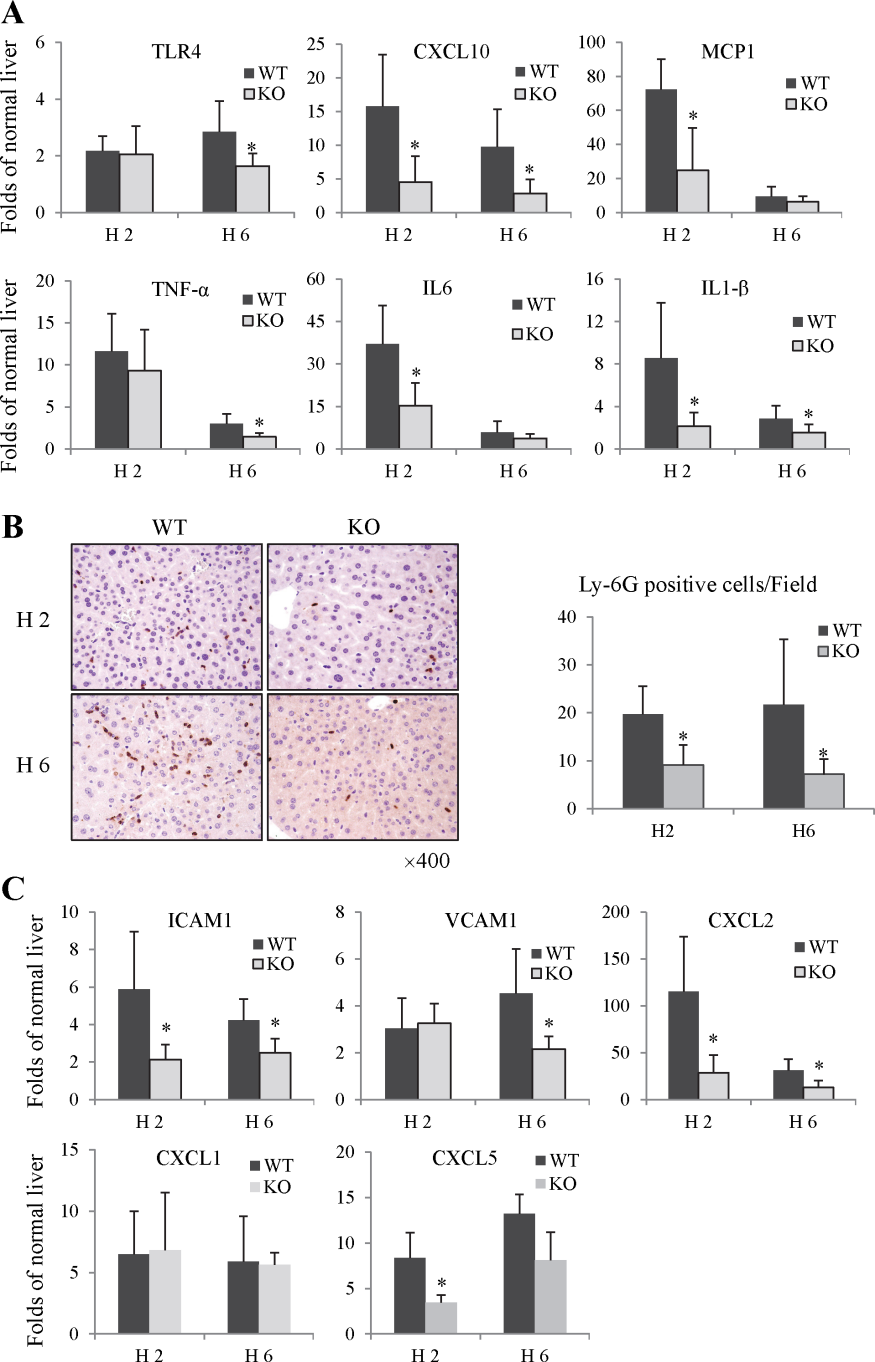
The knockout of Rap1 decreased hepatic inflammatory response and neutrophils recruitment in mouse IRI model (**A**) The hepatic mRNA levels of pro-inflammatory cytokines/chemokines were detected by RT-PCR. (**B**) Neutrophils infiltration was detected by IHC staining (Ly-6G). (**C**) The mRNA levels of neutrophils chemoattractants and adhesion factors were detected by RT-PCR. The gene expression levels were calculated as folds of normal liver. (*Compared to wild type group *P < 0.05*; *N* = 5–6/group).

The pro-inflammatory cytokines/chemokines have been implicated to play important roles in the pathogenesis of liver IRI. Compared to wild type group, the knockout of Rap1 reduced the mRNA levels of CXCL10 and TLR4 at 2 and 6 hours (Figure 3A). Furthermore, we also compared the expressions of TNF-α, IL-1β, IL6, and MCP1 between wild type and knockout group. The expressions of TNF-α, IL6, IL-1β and MCP1 in liver were found to be strikingly elevated after IRI in wild type group. On the other hand, decreased expressions of these pro-inflammatory cytokines/chemokines were observed in Rap1 knockout group (Figure 3A).

#### The knockout of Rap1 inhibited neutrophils recruitment and the expressions of neutrophils chemoattractants

During inflammation, circulating neutrophils are recruited to the site of inflammation and mediate the progression of inflammatory response. In Rap1 knockout mice model with IRI, hepatic neutrophils infiltration were decreased at 2 hours after reperfusion compared to these in wild type group (9.17 *vs* 19.73 cells/HPF). Furthermore, there were much less neutrophils in Rap1 knockout group at 6 hours after reperfusion compared to wild type group (7.2 *vs* 21.71 cells/HPF) (Figure 3B). The expressions of neutrophil chemoattractants and adhesion factors play key roles in regulating neutrophil migration and adhesion [22]. Compared to wild type group, the expressions of chemokine (C-X-C motif) ligand 2 (CXCL2), CXCL5, vascular cell adhesion molecule 1 (VCAM-1) and intercellular adhesion molecule 1 (ICAM1) were down-regulated in Rap1 knockout group (Figure 3C). However, there was no difference in CXCL1 expression between Rap1 wild type and knockout group.

### *In vitro* functional study

#### The knockout of Rap1 inhibited neutrophils migration and adhesion

**Figure 4:**
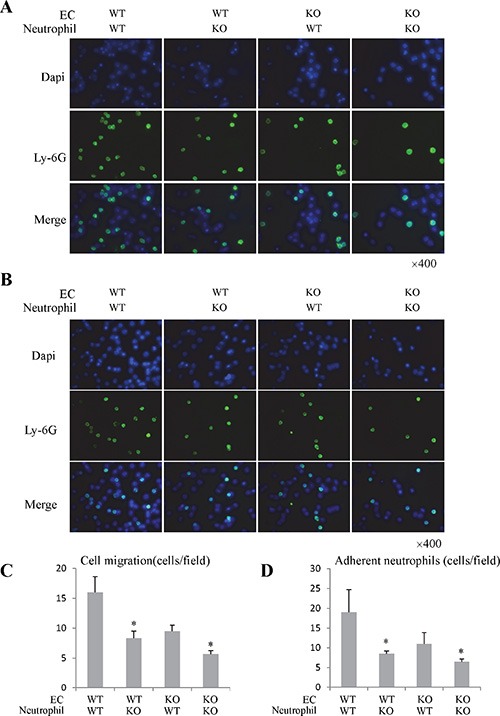
The knockout of Rap1 attenuated the neutrophils migration and adhesion activity *in vitro* study (**A**) The migration activity of neutrophils in response to LSECs was analyzed by neutrophils migration assay. (**B**) The neutrophils and LSECs adhesion was investigated through co-culture system. (**C**) The quantitative analysis of migrated neutrophils in response to LSECs. (**D**) The quantitative analysis of adherent neutrophils. (*Compared to wild type group *P < 0.05*).

In order to investigate the direct effect of Rap1 on neutrophils, we isolated the primary bone marrow neutrophils from Rap1 knockout and wild type mice. The isolated neutrophils were confirmed by flow cytometry and IF staining (Supplementary Figure 3A and 3B). Rap1 was up-regulated in neutrophils after activation (Supplementary Figure 4A). We next assessed neutrophils migration in response to liver LSECs and fMLP using transwell migration assay. The migration activity of Rap1 knockout neutrophils in response to LSECs and fMLP was lower than that of wild type neutrophils (Figure 4A and 4C; Supplementary Figure 5A). Similarly, neutrophils migration activity was suppressed in response to Rap1 knockout LSECs (Figure 4A and 4C). Furthermore, less neutrophils-LSEC adhesion was found in Rap1 knockout group compared to wild type group (Figure 4B and 4D).

#### The knockout of Rap1 down-regulated the expressions of chemoattractant receptor and F-actin in primary neutrophils

**Figure 5:**
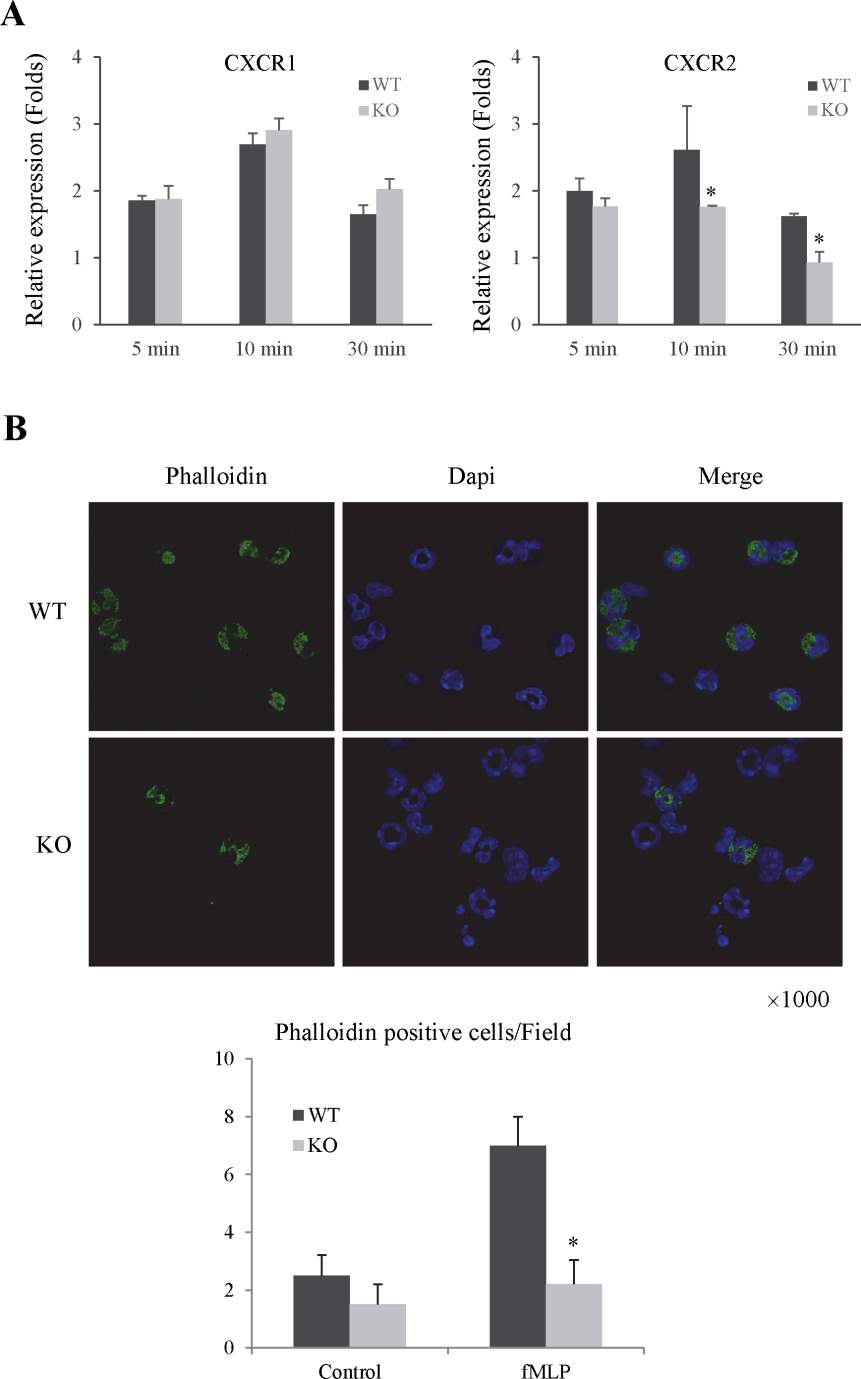
The knockout of Rap1 decreased the expressions of chemoattractant receptors and F-actin in primary neutrophils (**A**) The mRNA levels of chemoattractant receptors in neutrophils after fMLP activation were detected by RT-PCR. (**B**) The expression of F-Actin was detected by IF staining. (*Compared to wild type group *P < 0.05*).

In order to investigate the mechanism of Rap1 in neutrophils migration and adhesion, we compared the expression levels of chemoattractant receptors and F-action between Rap1 wild type and knockout neutrophils. The knockout of Rap1 decreased the expression of CXCR2 but not CXCR1 in primary neutrophils after fMLP and LPS activation (Figure 5A and Supplementary Figure 5B). Furthermore, the knockout of Rap1 also attenuated the expression of F-action in primary neutrophils after fMLP activation compared to wild type group (Figure 5B).

#### The knockout of Rap1 suppressed neutrophils induced inflammatory response and hepatocyte damage

**Figure 6:**
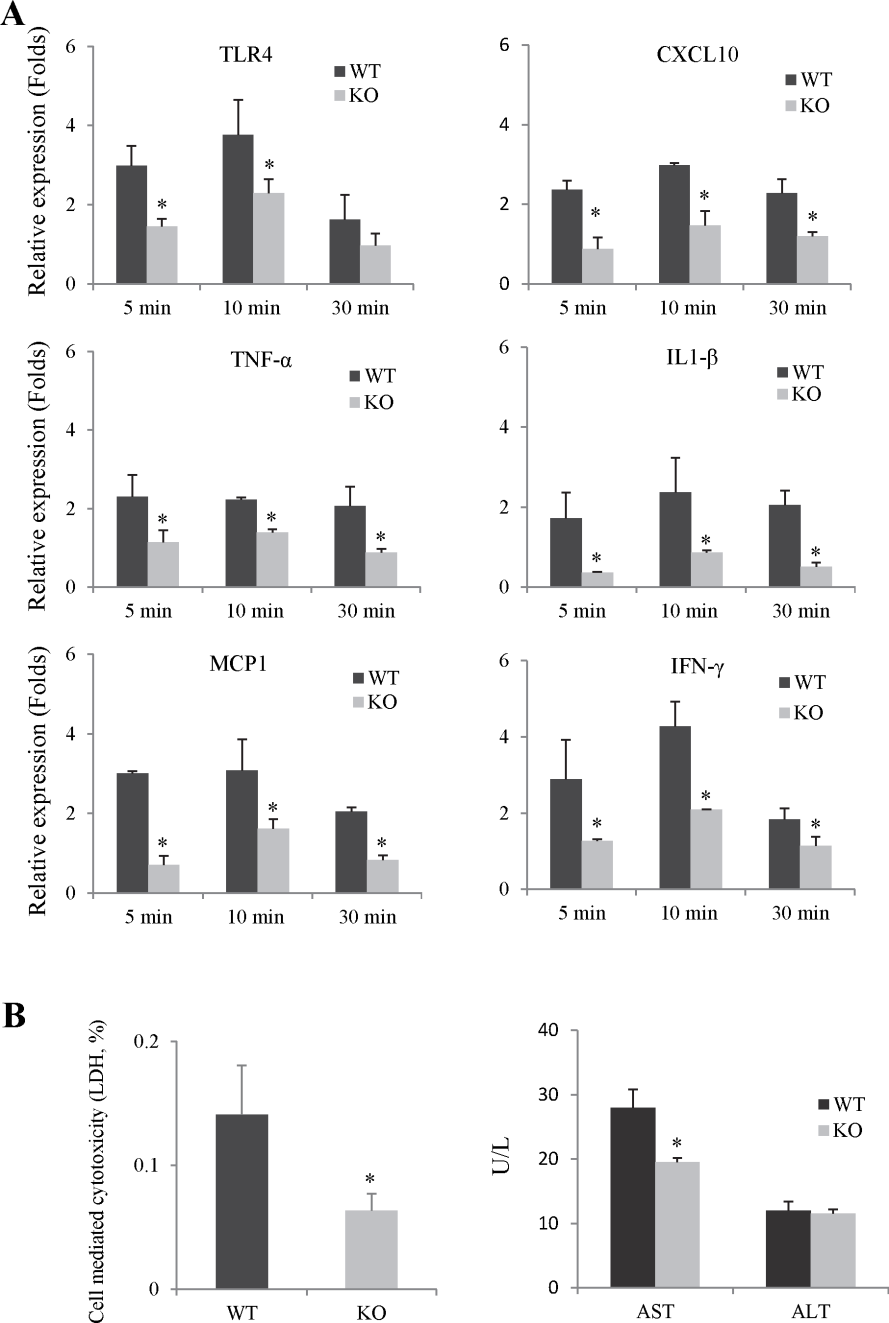
The knockout of Rap1 suppressed neutrophils induced inflammatory response and hepatocytes damage (**A**) The mRNA levels of pro-inflammatory cytokines/chemokines in primary neutrophils after fMLP activation were detected by RT-PCR. The gene expression levels were calculated as folds of untreated cells. (**B**) Neutrophils-induced hepatocytes injury was assessed by measurement of ALT, AST and LDH in the culture medium. (*Compared to wild type group *P < 0.05*).

The expressions of pro-inflammatory cytokines/chemokines in neutrophils after using fMLP and LPS activation were compared between Rap1 wild type and knockout group. The knockout of Rap1 decreased the expressions of pro-inflammatory cytokines/chemokines in primary neutrophils compared to wild type group (Figure 6A and Supplementary Figure 6A). Furthermore, we also tested the effect of Rap1 in neutrophils induced hepatocyte injury using the co-culture system. Lower LDH and AST levels were observed in Rap1 knockout group compared to wild type group (Figure 6B).

#### The knockout of Rap1 suppressed the activations of NF-κB and MAPK pathway

**Figure 7:**
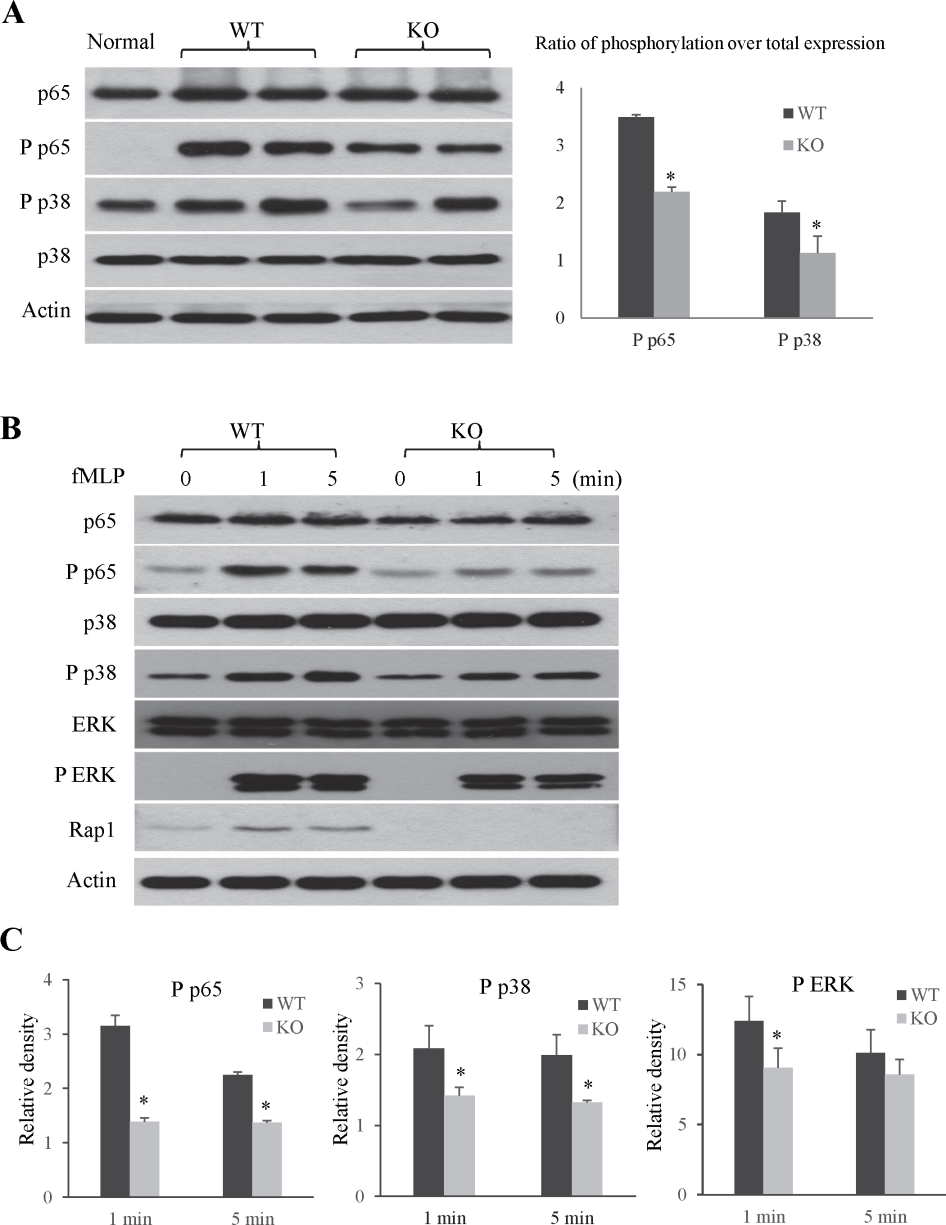
The knockout of Rap1 suppressed the activations of NF-κB and MAPK pathway (**A**) Activations of NF-κB and MAPK signaling pathway were detected in liver at 2 hours after liver IRI (*N* = 5–6/group). (**B**) Activations of NF-κB and MAPK signaling pathway in neutrophils after fMLP stimulation were detected by western blot. (**C**) Quantitative analysis of phosphorylation over total expression. (*Compared to wild type group *P < 0.05*).

To further explore the mechanism of Rap1 in regulating hepatic IRI both *in vivo* and *in vitro*, we detected the activations of inflammation-associated NF-κB and MAP kinases signaling pathway. In mouse model, the knockout of Rap1 suppressed the activations of phosphorylated p38 and p65 after hepatic IRI compared to wild type mice (Figure 7A). Similar results were also found in primary neutrophils. Rap1 deficiency suppressed the activation of phosphorylated p65 subunit of NF-κB in primary neutrophils. Furthermore, the knockout of Rap1 also suppressed the activations of phosphorylated ERK and p38 in primary neutrophils (Figure 7B and 7C, Supplementary Figure 6B).

## DISCUSSION

To our knowledge, this is the first report for the roles and mechanisms of Rap1 in hepatic IRI. We demonstrated that Rap1 was overexpressed in liver graft after liver transplantation, especially in LDLT. Consistent with our previous studies [23], hepatic injury makers such as hepatic inflammatory response after liver transplantation in LDLT group were higher compared to DDLT group. The result therefore suggested the positive correlation between Rap1 expression and acute-phase liver graft injury after transplantation. The results were also validated in rat liver transplantation and mouse hepatic IRI models. In functional studies, the knockout of Rap1 attenuated liver damage through inhibiting hepatic inflammatory response in both mouse IRI model *in vivo* and primary cells *in vitro*.

Hepatic IRI is a typical inflammatory response involving a complex web of interactions between various cellular and molecular signals. Macrophages and neutrophils play important roles in inflammatory response during liver IRI [5, 24]. In our study, over-expressed Rap1 was mainly found in neutrophils instead of macrophages in liver graft after human transplantation as well as cells after LPS or fMLP activation. Furthermore, the knockout of Rap1 suppressed neutrophil migration in mouse IRI model and decreased primary neutrophils migration activities in response to liver LSEC and fMLP. It suggested that over expression of Rap1 was neutrophils-dependent and Rap1 may induce hepatic inflammatory response through regulating neutrophils migration and function.

During inflammation, circulating neutrophils are recruited to the site of inflammation and then mediate the progression of inflammatory response. Recruitment and extravasation of neutrophils into the parenchyma are the first step and prerequisite for neutrophils cytotoxicity [10]. More evidences showed that a variety of inflammatory mediators (TNF-α, IL1β, CXCL1, and CXCL2) are important potent chemoattractants for neutrophils migration [25–27]. These mediators triggered the expressions of F-Actin and CXCR2 on the surface of neutrophils [28, 29]. The inhibition of CXC chemokines and CXC receptor attenuated hepatic neutrophils accumulation and liver injury during IRI. In the present study, the knockout of Rap1 significantly decreased expressions of adhesion molecules (ICAM1 and VCAM1) and chemoattractants (CXCL2, CXCL5) in mouse IRI model. In addition, the knockout of Rap1 also down-regulated the expressions of F-Actin and CXCR2 in primary neutrophils. These data indicated that Rap1 modulated neutrophils migration through regulating F-Actin expression and CXCL2/CXCR2 pathway. Furthermore, LSECs play important roles in regulating neutrophils migration and adhesion [30]. Our results showed that neutrophils migration and adhesion were suppressed in response to Rap1 knockout LSECs. The precise mechanism of Rap1 on regulating LSECs will be worthwhile for further studies.

Once entry into the parenchyma, neutrophils produce lots of reactive oxygen species and pro-inflammatory cytokines/chemokines, which not only directly cause hepatocytes damage but also aggravate inflammatory response [12, 31]. Rap1 acts as a regulator of the NF-κB signaling by promoting IKK-mediated phosphorylation of p65, leading to activation of the expression of NF-κB target genes [21]. In this study, the knockout of Rap1 decreased production of pro-inflammatory cytokines/chemokines in both mouse IRI model and primary neutrophils. In addition, the knockout of Rap1 suppressed the activations of NF-κB and MAPK signaling pathway in mouse hepatic IRI model and primary neutrophils. The results indicated that Rap1 regulated the neutrophils induced inflammatory response via NF-κB and MAPK signaling pathway.

In summary, we firstly explored the roles and mechanisms of Rap1 in hepatic IRI. The knockout of Rap1 attenuated hepatic IRI not only through inhibiting neutrophils migration, but also suppressing the function of neutrophils through NF-κB and MAPK signaling pathway. Due to hepatic IRI is a complex process and multiple pathways can be involved, further studies for the mechanism of Rap1 in regulating liver IRI will be needed.

## MATERIALS AND METHODS

### Clinical samples

From February 2008 to November 2011, 53 patients (LDLT: *n* = 33; DDLT: *n* = 20) received liver transplantation in Department of Surgery, Queen Mary Hospital, The University of Hong Kong, were included in the current study. The human liver graft biopsy samples were collected at 2 hours after portal vein reperfusion during the recipient's operation. The study protocol was approved by the Institutional Review Board of The University of Hong Kong (IRB approval number: UW_11-099). Informed consent was obtained from each patient.

### Animal models

The information of Rap1 knockout mice were described in our previous paper [32]. Sprague Dawley (SD) rats were purchased from the Laboratory Animal Unit, The University of Hong Kong (HKULAU). The study had been licensed according to Animal Ordinance Chapter 340 by the Department of Health, Hong Kong Special Administrative Region (ref.: (14–613) in DH/HA&P/8/2/3 Pt. 63).

### Mouse hepatic IRI model

Rap1 knockout and wild type mice were subjected to major hepatectomy (left and caudate lobes) and partial hepatic IRI (1 hour ischemia of right and triangle lobes) to mimic the clinical situation [24]. The mice were sacrificed at 2 hours, 6 hours, and 24 hours after reperfusion and liver samples were collected for further studies. The serum was collected for detecting the serum aspartate aminotransferase (AST) and alanine transaminase (ALT) levels.

### Rat orthotopic liver transplantation

Rat orthotopic liver transplantation was conducted in two group (i) whole graft group (Number of recipients = 6), (ii) small-for-size (SFS) graft group (Number of recipients = 6). Liver tissues were sampled at 4 hours after reperfusion for further analysis. The details of surgical procedure were described previously [24].

### Immunohistochemical and immunofluorescence staining

The expression of Rap1, neutrophil, and F-actin were detected by immunohistochemical (IHC) or immunofluorescence (IF) staining. The details of staining were described previously [33].

### Isolation and activation of mouse bone marrow neutrophil

Bone marrow neutrophils were isolated from Rap1 knockout and wild type mice [34]. Isolated neutrophils were treated with LPS (1 μg/ml) or *N*-Formylmethionyl-leucyl-phenylalanine (fMLP, 1 μM) and then detected the expressions of inflammatory cytokines/chemokines and activations of NF-κB and MAPK pathway.

### Isolation of mouse primary hepatocytes and liver sinusoidal endothelial cells (LSECs)

Hepatocytes and LSECs were isolated from mice using two-step collagenase perfusion [35]. In brief, liver was perfused with Ca^2+^ and Mg^2+^ free HBSS (Invitrogen) for 5 minutes, followed by perfusion with 0.04% collagenase IV (Sigma) in HBSS for 10 minutes at 37°C. And then the suspension was centrifuged at 35 g for 2 minutes. To hepatocyte, pellets were washed three times with HBSS and overlay the 60% percoll, and then centrifuged 3000 rpm for 10 minutes. For LSECs, supernatants were centrifuged at 1350 g for 10 minutes, and then resuspend pellet in 10 ml preservation buffer. The cells were load on top of 25%/50% percoll gradient and centrifuged 1350 g for 30 minutes. The non-parenchymal cells (NPC) were collected from the interface between the two density cushions followed by removing the Kupffer cell by selective adherence.

### Neutrophil migration assay

Confluent LSECs plated onto 24-well plates were treated with 20 ng/ml TNF-α for 6 hours. After that, neutrophils (1 × 10^6^/100 ul) from Rap1 knockout and wild mice were placed in the upper chamber and incubated for 2 h at 37°C. Furthermore, we also investigated the migration activity of neutrophil in response to the chemoattractants fMLP. The fMLP were placed in the lower chambers while neutrophils were placed in the upper chamber for incubating of 2 hours at 37°C. The plate was centrifuged after the filter was removed. The numbers of cells transmigrated to the lower compartments were stained with Ly-6G and counted using an inverted microscope.

### Neutrophil and endothelial cell adhesion

Confluent LSECs plated onto 24-well plates were treated with 20 ng/ml TNF-α. Six hours later, neutrophils were added to the LSECs. The plate was gently centrifuged at 200 g for 2 minutes and neutrophils were allowed to adhere for 15 minutes. Non-adherent neutrophils were washed and the cells were fixed with 10% buffered formalin. The adhered neutrophils were stained with Ly-6G and counted using an inverted microscope.

### Assessment of neutrophils-mediated hepatocyte injury

We next tested the effect of Rap1 on neutrophils induced hepatocyte injury using the co-culture system. To mimic the condition during hepatic IRI *in vivo*, hepatocytes were cultured for 6 hours under hypoxic conditions and then co-cultured with neutrophils from Rap1 knockout or wild type mice. After 2 hours co-culture, toxicity was assessed by measurement of ALT, AST and lactate dehydrogenase (LDH) activity in the culture medium.

### Detection of gene expression by real-time RT-PCR

RT-PCR was done with a modified version of a previous method [24]. Gene expression levels were expressed as the folds relative to the normal liver or untreated cells.

### Measurement of protein levels by western blot

Western blot was done with a modified version of a previous method [24]. The protein expression levels were calculated as folds of normal liver or untreated cells. β-Actin, anti-NF-κB, anti-Rap1, anti-JNK, anti-ERK and anti-p38 antibodies were purchased from Cell Signaling Technology or ABCAM.

### Statistics and data analyses

Continuous variables were expressed as average with standard deviation (SD). *T* test was used for statistical comparison. *P* < 0.05 was considered statistically significant. Calculations were performed by using the SPSS computer software version 16.

## SUPPLEMENTARY MATERIALS FIGURES



## Abbreviations

Rap1: Repressor and activator protein
NF-κB: nuclear factor-κB
IRI: ischemia reperfusion injury
LSECs: liver sinusoidal endothelial cells
TLR4: Toll-like receptor 4
CXCL10: C-X-C motif chemokine 10
AST: aspartate aminotransferase
ALT: alanine transaminase
IHC: immunohistochemical
IF: immunofluorescence
LDH: lactate dehydrogenase
SFS: small-for-size
HO1: heme oxygenase
Nrf2: nuclear factor erythroid-derived 2-like 2
CXCL: chemokine (C-X-C motif) ligand
VCAM-1: vascular cell adhesion molecule 1
ICAM1: intercellular adhesion molecule 1.
