# Supplementary material for: Repressor and activator protein accelerates hepatic ischemia reperfusion injury by promoting neutrophil inflammatory response
Source: Oncotarget. 2016 Mar 30;7(19):27711–23. doi: 10.18632/oncotarget.8509 (PMC5053682; doi:10.18632/oncotarget.8509)
Supplement: Supplementary file 1 [file oncotarget-07-27711-s001.pdf]

Repressor and activator protein accelerates hepatic ischemia reperfusion injury by promoting neutrophil inflammatory response

Supplementary Materials

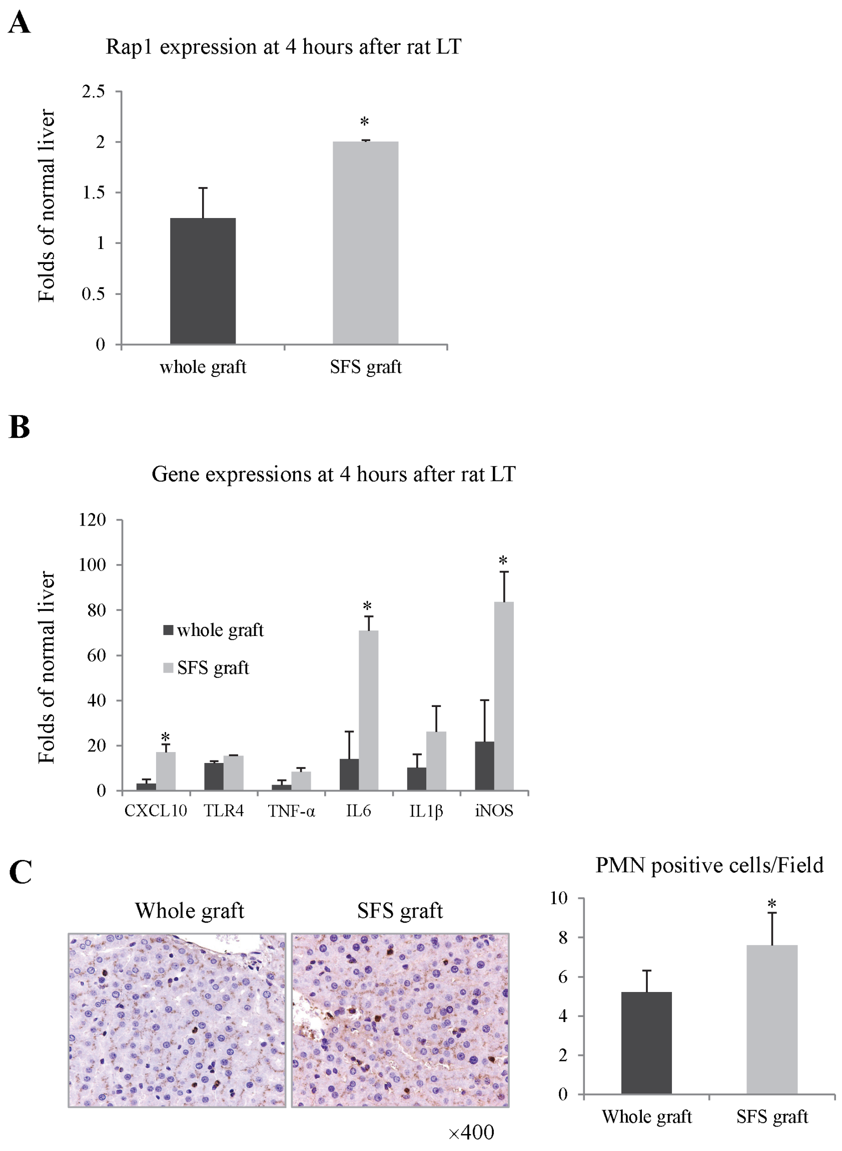

Supplementary Figure S1: Over expression of Rap1 was associated with severe hepatic inflammatory response after rat liver transplantation. (**A**) The intragraft mRNA level of Rap1 at 4 hours after rat transplantation. (**B**) The intragraft expressions of pro-inflammatory cytokines/chemokines at 4 hours after rat transplantation. (**C**) Neutrophils infiltration were detected by IHC staining. (Compared to whole graft $*P < 0.05$).

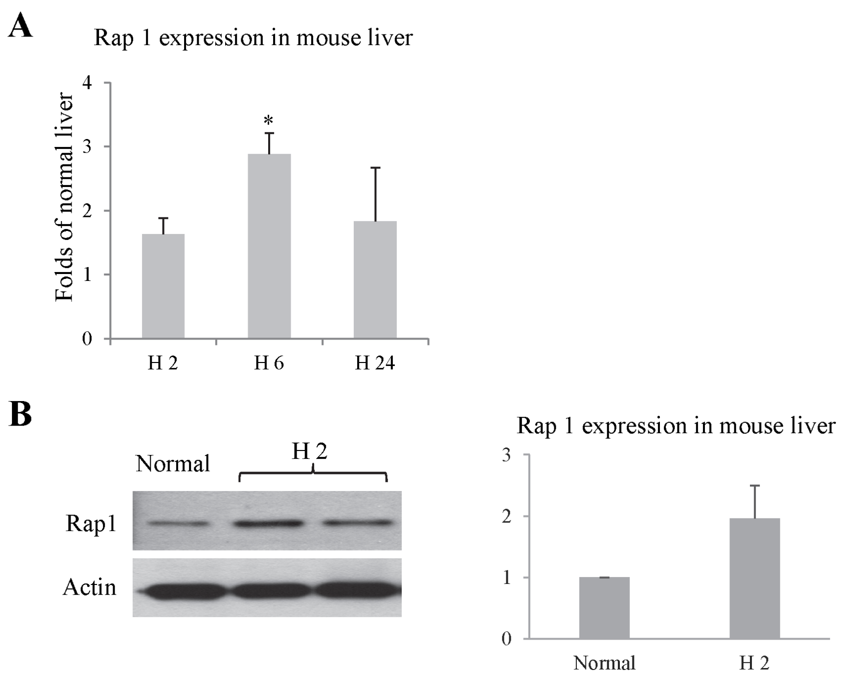

Supplementary Figure S2: Rap1 was over-expressed in mouse liver after hepatic IRI. (**A**) The mRNA level of Rap1 in mouse liver after hepatic IRI was detected by RT-PCR. (**B**) The protein level of Rap1 in mouse liver after hepatic IRI was detected by western blot. (Compared to H2 *$P < 0.05$).

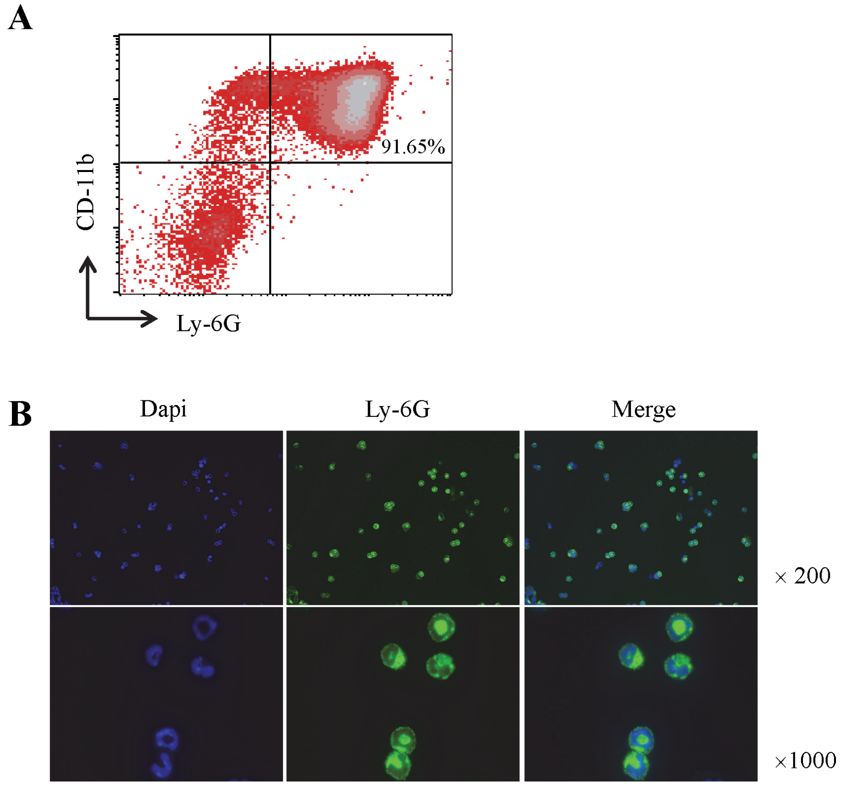

Supplementary Figure S3: Primary neutrophils were isolated from mouse bone marrow. (**A**) The isolated neutrophils were confirmed by flow cytometry. (**B**) The isolated neutrophils were staining with Ly-6G antibody.

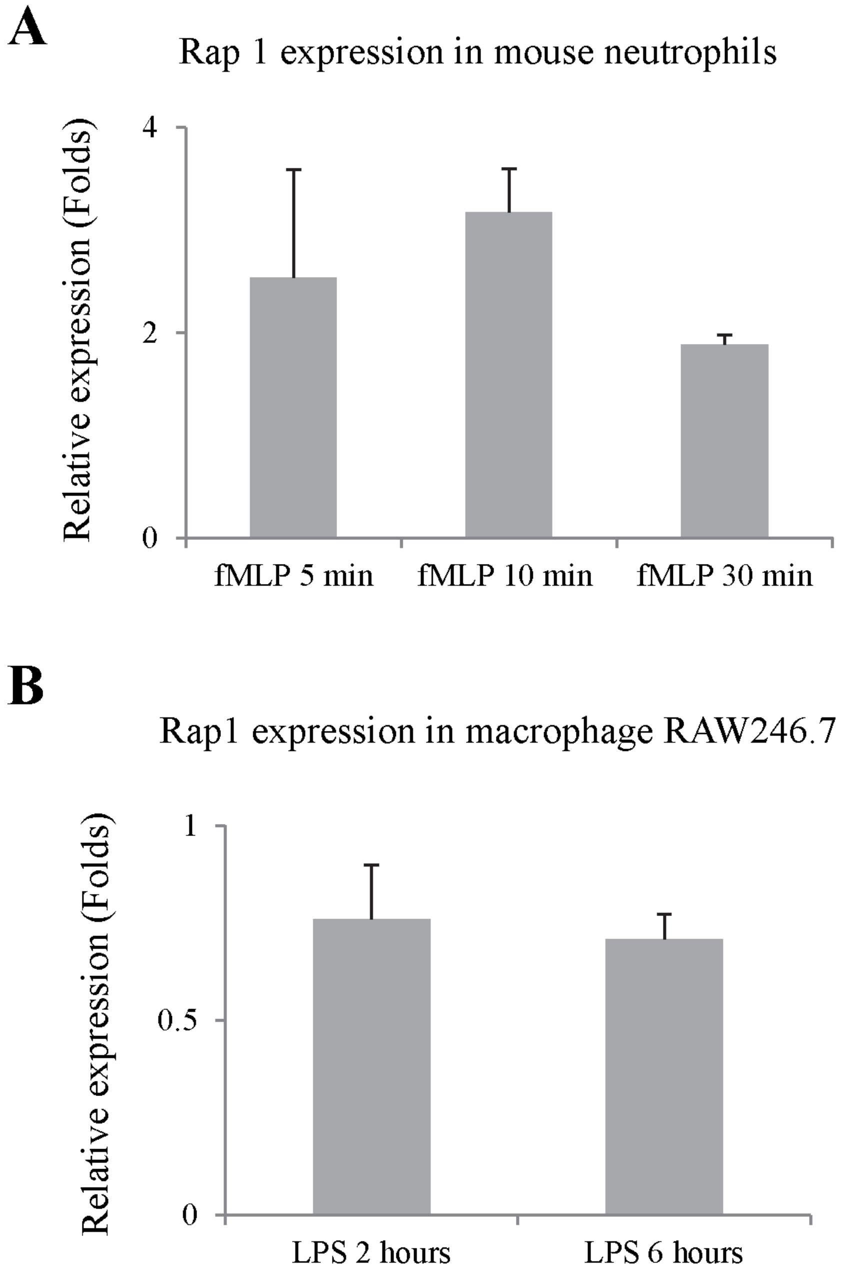

Supplementary Figure S4: The expressions of Rap1 in neutrophils and macrophages were detected. (**A**) The mRNA level of Rap1 in mouse primary neutrophils after fMLP activation was detected by RT-PCR. (**B**) The mRNA level of Rap1 in macrophages after LPS activation was detected by RT-PCR. Gene expression levels were expressed as the folds relative to untreated cells.

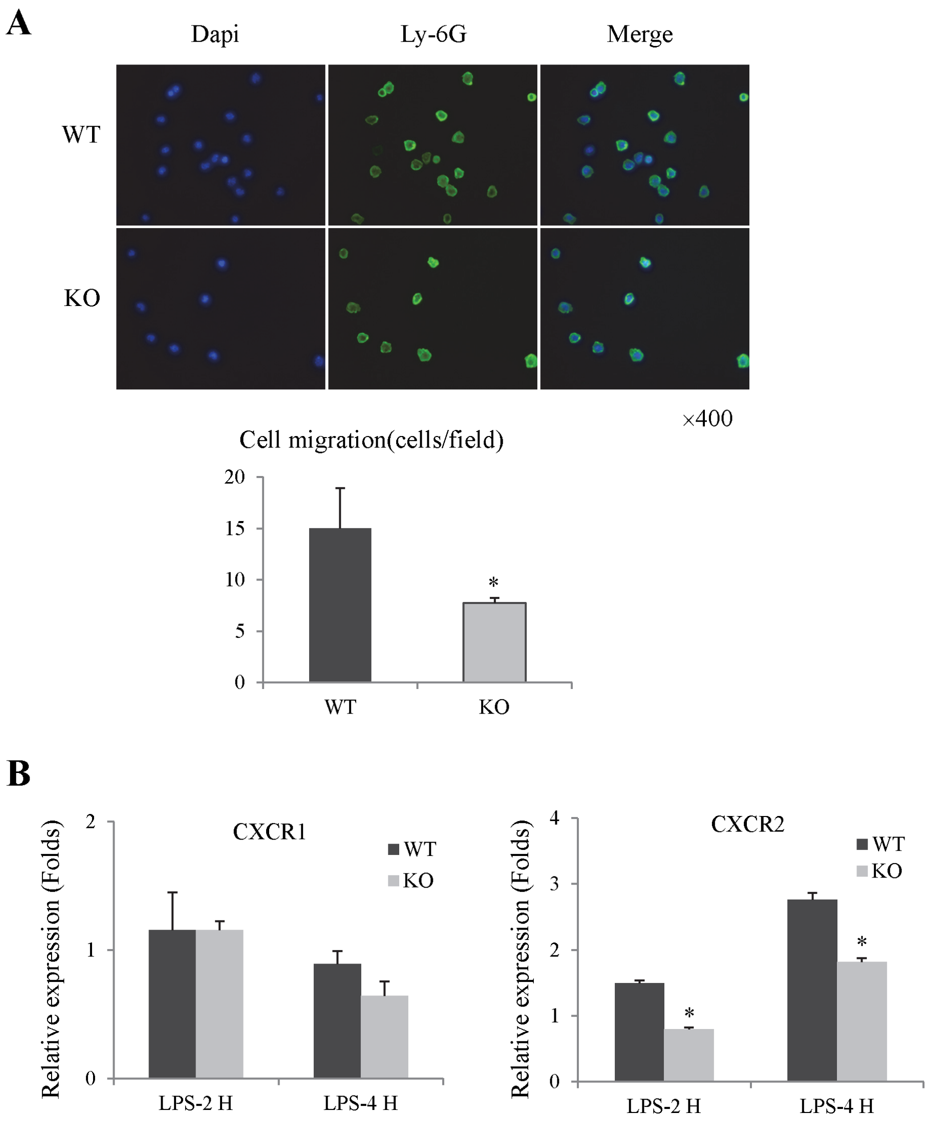

Supplementary Figure S5: The knockout of Rap1 suppressed neutrophils migration activity. (**A**) Neutrophils migration in response to fMLP were detected by IHC staining (Ly-6G). (**B**) The mRNA levels of neutrophils surface receptors after LPS activation were detected by RT-PCR. (*Compared to wild type group $P < 0.05$).

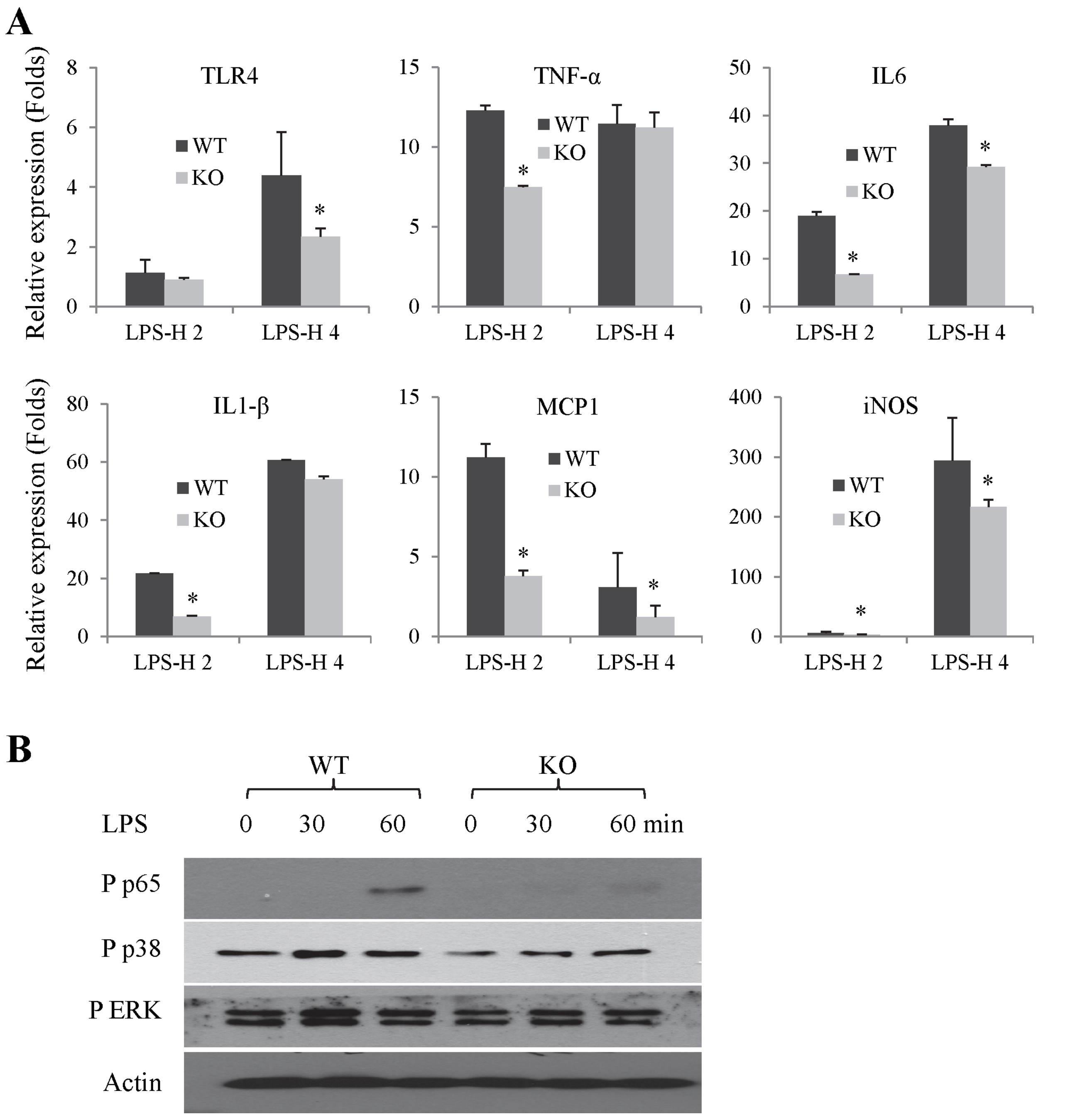

Supplementary Figure S6: The knockout of Rap1 suppressed expressions of pro-inflammatory cytokines/chemokines and activations of NF-κB and MAPK pathway in primary neutrophils after LPS activation. (**A**) The mRNA levels of pro-inflammatory cytokines/chemokines in primary neutrophils were detected by RT-PCR. The gene expression levels were calculated as folds of untreated cells. (**B**) Activations of NF-κB and MAPK signaling pathway in primary neutrophils were detected by western blot. (*Compared to wild type group $P < 0.05$)
